# *trans*-Diaqua­bis[5-(1*H*-imidazol-4-yl-κ*N* ^3^)-1*H*-tetra­zolato-κ*N* ^1^]zinc(II)

**DOI:** 10.1107/S1600536809013567

**Published:** 2009-04-22

**Authors:** Hong Zhao, Jie Xiao

**Affiliations:** a Ordered Matter Science Research Centre, College of Chemistry and Chemical Engineering, Southeast University, Nanjing 210096, People’s Republic of China

## Abstract

In the title complex, [Zn(C_4_H_3_N_6_)_2_(H_2_O)_2_], the metal centre lies on an inversion centre and displays a distorted octa­hedral ZnN_4_O_2_ coordination geometry. The organic ligand is not planar; the dihedral angle between the imidazole and tetra­zole rings is 8.39 (9)°. An extended network of inter­molecular N—H⋯N and O—H⋯N hydrogen bonds stabilizes the crystal structure.

## Related literature

For the synthesis and properties of tetra­zole compounds, see: Demko & Sharpless (2001 ▶, 2002 ▶); Zhao *et al.* (2008 ▶).
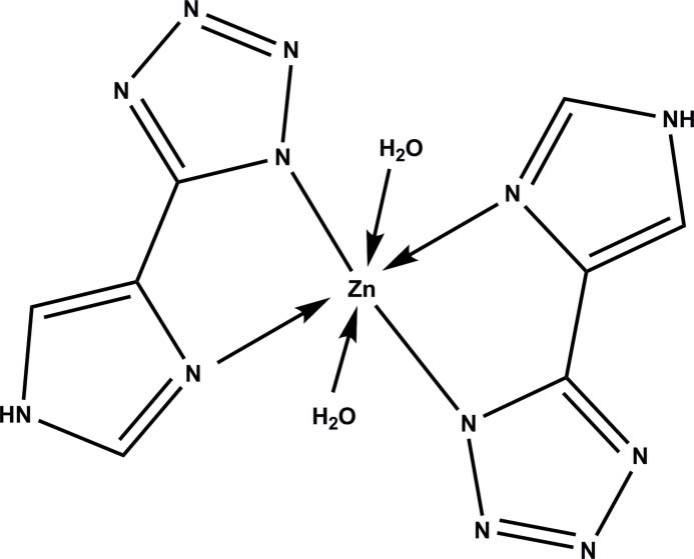


## Experimental

### 

#### Crystal data

[Zn(C_4_H_3_N_6_)_2_(H_2_O)_2_]
*M* *_r_* = 371.65
Monoclinic, 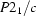

*a* = 5.9068 (10) Å
*b* = 17.408 (3) Å
*c* = 7.091 (2) Å
β = 110.70 (2)°
*V* = 682.1 (3) Å^3^
*Z* = 2
Mo *K*α radiation
μ = 1.84 mm^−1^
*T* = 291 K
0.20 × 0.18 × 0.15 mm

#### Data collection

Rigaku SCXmini diffractometer
Absorption correction: multi-scan (*CrystalClear*; Rigaku, 2005 ▶) *T*_min_ = 0.753, *T*_max_ = 0.762
6793 measured reflections
1555 independent reflections
1429 reflections with *I* > 2σ(*I*)
*R*_int_ = 0.025

#### Refinement

*R*[*F* ^2^ > 2σ(*F* ^2^)] = 0.028
*wR*(*F* ^2^) = 0.113
*S* = 1.30
1555 reflections
106 parameters
H-atom parameters constrained
Δρ_max_ = 0.59 e Å^−3^
Δρ_min_ = −0.64 e Å^−3^

### 

Data collection: *CrystalClear* (Rigaku, 2005 ▶); cell refinement: *CrystalClear*; data reduction: *CrystalClear*; program(s) used to solve structure: *SHELXS97* (Sheldrick, 2008 ▶); program(s) used to refine structure: *SHELXL97* (Sheldrick, 2008 ▶); molecular graphics: *SHELXTL/PC* (Sheldrick, 2008 ▶); software used to prepare material for publication: *SHELXTL/PC*.

## Supplementary Material

Crystal structure: contains datablocks I, global. DOI: 10.1107/S1600536809013567/rz2306sup1.cif

Structure factors: contains datablocks I. DOI: 10.1107/S1600536809013567/rz2306Isup2.hkl

Additional supplementary materials:  crystallographic information; 3D view; checkCIF report

## Figures and Tables

**Table 1:** Hydrogen-bond geometry (Å, °)

*D*—H⋯*A*	*D*—H	H⋯*A*	*D*⋯*A*	*D*—H⋯*A*
N2—H2*A*⋯N6^i^	0.86	2.01	2.803 (3)	153
O1—H1*B*⋯N5^ii^	0.86	2.00	2.837 (3)	164
O1—H1*A*⋯N4^iii^	0.79	2.08	2.841 (2)	164

Symmetry codes: (i) 
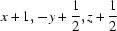
; (ii) 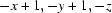; (iii) 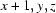.

**Fig. 1.**
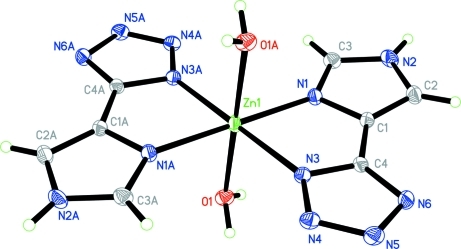
The molecular structure of the title compound, showing the atomic numbering scheme. Displacement ellipsoids are drawn at the 30% probability level. Atoms labelled with suffix A are generated by the symmetry operation (2-x, 1-y, 1-z).

**Fig. 2.**
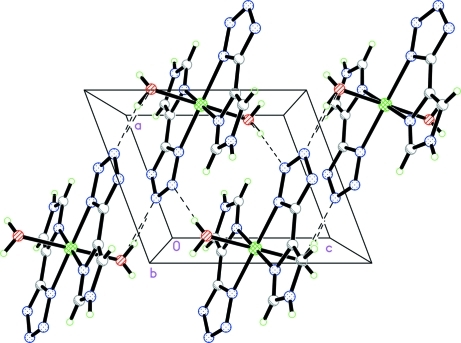
Packing diagram of the title compound viewed along the b axis. Intermolecular hydrogen bonds are shown as dashed lines.

## supplementary crystallographic information

### Comment

Tetrazole ligands have found a wide range of applications in medicine chemistry, coordination chemistry and material chemistry (Demko & Sharpless, 2001). Recently, the tetrazole synthesis in water has attracted intense attention. For example, a safe, convenient, and environmentally friendly procedure for the synthesis of 5-substituted 1*H*-tetrazoles, which were prepared by the addition of azides to nitriles in water using zinc salts as catalysts, has been reported (Demko & Sharpless, 2002). Our group has been interested in the construction of novel supramolecular motifs through *in situ* hydrothermal reactions (Zhao *et al.*, 2008). In particular, we have combined metal salts with potentially bridging organic ligands under hydrothermal conditions to produce a range of new materials in order to investigate the Demko-Sharpless reaction. Herein we report on the synthesis and structure of the title compound, which was obtained by the hydrothermal reaction of ZnCl_2_ with (4-cyano)-imidazole and NaN_3_ in water.

Figure 1 shows the monomeric complex molecule along with the atom-labelling scheme. The zinc(II) metal lies on an inversion centre, and displays a distorted octahedral coordination geometry provided by the N atoms of two chelating ligands at the equatorial plane and by the oxygen atoms of two trans-arranged water molecules at the axial positions. The bond distances and angles within the coordination octahedron have normal values. The organic ligand is not planar, the dihedral angle formed by the imidazole and tetrazole rings is 8.39 (9)°. The five-membered chelating ring assumes an approximately planar conformation (maximum deviation 0.030 (2) Å for atom C4). The crystal structure is stabilized by intermolecular N—H···N and O—H···N hydrogen bonds (Table 1), forming an extended three-dimensional network (Fig. 2).

### Experimental

Colourless single crystals of title compound were obtained by hydrothermal treatment of ZnCl_2_ (1 mmol), NaN_3_ (3 mmol), (4-cyano)-imidazole (1 mmol) and water (7 ml) over 1 day at 398 K. Yield: 53% (based on ZnCl_2_).

### Refinement

The water H atoms were located from a difference Fourier map but not refined [*U*_iso_(H) = 1.5 *U*_eq_(O)]. All other H atoms were placed at calculated positions and refined as riding, with C—H = 0.93 Å, N—H = 0.86 Å, and with *U*_iso_(H) = 1.2 *U*_eq_(C, N).

### Crystal data

**Table:**

[Zn(C_4_H_3_N_6_)_2_(H_2_O)_2_]	*F*(000) = 376
*M**_r_* = 371.65	*D*_x_ = 1.809 Mg m^−^^3^
Monoclinic, *P*2_1_/*c*	Mo *K*α radiation, λ = 0.71073 Å
Hall symbol: -P 2ybc	Cell parameters from 2098 reflections
*a* = 5.9068 (10) Å	θ = 2.3–27.5°
*b* = 17.408 (3) Å	µ = 1.84 mm^−^^1^
*c* = 7.091 (2) Å	*T* = 291 K
β = 110.70 (2)°	Prism, colourless
*V* = 682.1 (3) Å^3^	0.20 × 0.18 × 0.15 mm
*Z* = 2	

### Data collection

**Table:**

Rigaku SCXmini diffractometer	1555 independent reflections
Radiation source: fine-focus sealed tube	1429 reflections with *I* > 2σ(*I*)
graphite	*R*_int_ = 0.025
Detector resolution: 13.6612 pixels mm^-1^	θ_max_ = 27.5°, θ_min_ = 2.3°
ω scans	*h* = −7→7
Absorption correction: multi-scan (*CrystalClear*; Rigaku, 2005)	*k* = −22→22
*T*_min_ = 0.753, *T*_max_ = 0.762	*l* = −9→9
6793 measured reflections	

### Refinement

**Table:**

Refinement on *F*^2^	Primary atom site location: structure-invariant direct methods
Least-squares matrix: full	Secondary atom site location: difference Fourier map
*R*[*F*^2^ > 2σ(*F*^2^)] = 0.028	Hydrogen site location: inferred from neighbouring sites
*wR*(*F*^2^) = 0.113	H-atom parameters constrained
*S* = 1.30	*w* = 1/[σ^2^(*F*_o_^2^) + (0.0683*P*)^2^ + 0.021*P*] where *P* = (*F*_o_^2^ + 2*F*_c_^2^)/3
1555 reflections	(Δ/σ)_max_ < 0.001
106 parameters	Δρ_max_ = 0.59 e Å^−^^3^
0 restraints	Δρ_min_ = −0.64 e Å^−^^3^

### Special details

**Table:**

Geometry. All e.s.d.'s (except the e.s.d. in the dihedral angle between two l.s. planes) are estimated using the full covariance matrix. The cell e.s.d.'s are taken into account individually in the estimation of e.s.d.'s in distances, angles and torsion angles; correlations between e.s.d.'s in cell parameters are only used when they are defined by crystal symmetry. An approximate (isotropic) treatment of cell e.s.d.'s is used for estimating e.s.d.'s involving l.s. planes.
Refinement. Refinement of *F*^2^ against ALL reflections. The weighted *R*-factor *wR* and goodness of fit *S* are based on *F*^2^, conventional *R*-factors *R* are based on *F*, with *F* set to zero for negative *F*^2^. The threshold expression of *F*^2^ > σ(*F*^2^) is used only for calculating *R*-factors(gt) *etc*. and is not relevant to the choice of reflections for refinement. *R*-factors based on *F*^2^ are statistically about twice as large as those based on *F*, and *R*- factors based on ALL data will be even larger.

### Fractional atomic coordinates and isotropic or equivalent isotropic displacement parameters (Å^2^)

**Table:**

	*x*	*y*	*z*	*U*_iso_*/*U*_eq_	
Zn1	1.0000	0.5000	0.5000	0.02202 (18)	
C1	0.9503 (4)	0.34932 (12)	0.3008 (3)	0.0212 (4)	
C2	1.0396 (4)	0.27845 (13)	0.2890 (4)	0.0287 (5)	
H2	0.9581	0.2376	0.2090	0.034*	
C3	1.3185 (4)	0.34887 (13)	0.5063 (4)	0.0262 (5)	
H3	1.4657	0.3635	0.6021	0.031*	
C4	0.7145 (4)	0.38475 (12)	0.2122 (3)	0.0207 (4)	
N1	1.1269 (3)	0.39332 (10)	0.4390 (3)	0.0223 (4)	
N2	1.2733 (4)	0.27947 (11)	0.4186 (3)	0.0292 (4)	
H2A	1.3746	0.2421	0.4404	0.035*	
N3	0.6743 (3)	0.45320 (10)	0.2795 (3)	0.0208 (4)	
N4	0.4411 (3)	0.46893 (11)	0.1772 (3)	0.0242 (4)	
N5	0.3469 (3)	0.41230 (11)	0.0538 (3)	0.0272 (4)	
N6	0.5162 (3)	0.35808 (11)	0.0704 (3)	0.0258 (4)	
O1	1.0962 (3)	0.56118 (9)	0.2680 (2)	0.0285 (4)	
H1B	0.9793	0.5753	0.1617	0.043*	
H1A	1.1972	0.5434	0.2327	0.043*	

### Atomic displacement parameters (Å^2^)

**Table:**

	*U*^11^	*U*^22^	*U*^33^	*U*^12^	*U*^13^	*U*^23^
Zn1	0.0195 (3)	0.0171 (2)	0.0251 (3)	0.00042 (11)	0.00252 (17)	−0.00441 (12)
C1	0.0214 (10)	0.0190 (10)	0.0211 (10)	−0.0010 (7)	0.0049 (8)	−0.0013 (8)
C2	0.0301 (12)	0.0212 (11)	0.0323 (12)	0.0020 (9)	0.0081 (9)	−0.0019 (9)
C3	0.0198 (10)	0.0270 (11)	0.0287 (11)	0.0035 (8)	0.0045 (9)	0.0030 (9)
C4	0.0210 (10)	0.0175 (9)	0.0216 (10)	−0.0022 (7)	0.0052 (8)	0.0008 (8)
N1	0.0186 (9)	0.0194 (8)	0.0256 (9)	0.0007 (7)	0.0039 (7)	−0.0019 (7)
N2	0.0271 (10)	0.0233 (9)	0.0356 (11)	0.0098 (8)	0.0089 (8)	0.0035 (8)
N3	0.0169 (9)	0.0214 (9)	0.0217 (9)	0.0030 (7)	0.0037 (7)	−0.0004 (7)
N4	0.0177 (9)	0.0277 (10)	0.0254 (10)	0.0021 (7)	0.0051 (7)	0.0028 (8)
N5	0.0192 (9)	0.0297 (10)	0.0286 (10)	−0.0008 (7)	0.0031 (7)	0.0028 (8)
N6	0.0215 (9)	0.0214 (9)	0.0282 (10)	−0.0031 (7)	0.0011 (7)	−0.0021 (8)
O1	0.0226 (8)	0.0344 (9)	0.0259 (8)	0.0058 (6)	0.0055 (6)	0.0016 (7)

### Geometric parameters (Å, °)

**Table:**

Zn1—N1^i^	2.1042 (18)	C3—N1	1.313 (3)
Zn1—N1	2.1042 (18)	C3—N2	1.342 (3)
Zn1—N3^i^	2.1641 (19)	C3—H3	0.9300
Zn1—N3	2.1641 (19)	C4—N6	1.329 (3)
Zn1—O1	2.1966 (17)	C4—N3	1.336 (3)
Zn1—O1^i^	2.1966 (17)	N2—H2A	0.8600
C1—C2	1.356 (3)	N3—N4	1.339 (3)
C1—N1	1.383 (3)	N4—N5	1.305 (3)
C1—C4	1.448 (3)	N5—N6	1.350 (3)
C2—N2	1.362 (3)	O1—H1B	0.8585
C2—H2	0.9300	O1—H1A	0.7874
			
N1^i^—Zn1—N1	180.0	N1—C3—N2	110.9 (2)
N1^i^—Zn1—N3^i^	79.02 (7)	N1—C3—H3	124.5
N1—Zn1—N3^i^	100.98 (7)	N2—C3—H3	124.5
N1^i^—Zn1—N3	100.98 (7)	N6—C4—N3	111.23 (19)
N1—Zn1—N3	79.02 (7)	N6—C4—C1	129.4 (2)
N3^i^—Zn1—N3	180.00 (8)	N3—C4—C1	119.34 (19)
N1^i^—Zn1—O1	86.07 (7)	C3—N1—C1	105.58 (19)
N1—Zn1—O1	93.93 (7)	C3—N1—Zn1	140.26 (16)
N3^i^—Zn1—O1	87.66 (7)	C1—N1—Zn1	113.59 (14)
N3—Zn1—O1	92.34 (7)	C3—N2—C2	108.22 (18)
N1^i^—Zn1—O1^i^	93.93 (7)	C3—N2—H2A	125.9
N1—Zn1—O1^i^	86.07 (7)	C2—N2—H2A	125.9
N3^i^—Zn1—O1^i^	92.34 (7)	C4—N3—N4	105.51 (17)
N3—Zn1—O1^i^	87.66 (7)	C4—N3—Zn1	111.67 (14)
O1—Zn1—O1^i^	180.00 (7)	N4—N3—Zn1	142.77 (14)
C2—C1—N1	109.58 (19)	N5—N4—N3	108.77 (18)
C2—C1—C4	134.1 (2)	N4—N5—N6	110.06 (17)
N1—C1—C4	116.14 (18)	C4—N6—N5	104.42 (19)
C1—C2—N2	105.7 (2)	Zn1—O1—H1B	117.1
C1—C2—H2	127.2	Zn1—O1—H1A	118.2
N2—C2—H2	127.2	H1B—O1—H1A	107.4

Symmetry codes: (i) −*x*+2, −*y*+1, −*z*+1.

### Hydrogen-bond geometry (Å, °)

**Table:**

*D*—H···*A*	*D*—H	H···*A*	*D*···*A*	*D*—H···*A*
N2—H2A···N6^ii^	0.86	2.01	2.803 (3)	153
O1—H1B···N5^iii^	0.86	2.00	2.837 (3)	164
O1—H1A···N4^iv^	0.79	2.08	2.841 (2)	164

Symmetry codes: (ii) *x*+1, −*y*+1/2, *z*+1/2; (iii) −*x*+1, −*y*+1, −*z*; (iv) *x*+1, *y*, *z*.
